# Enhancing Quality of Fresh Vegetables Through Salinity Eustress and Biofortification Applications Facilitated by Soilless Cultivation

**DOI:** 10.3389/fpls.2018.01254

**Published:** 2018-08-22

**Authors:** Youssef Rouphael, Marios C. Kyriacou

**Affiliations:** ^1^Department of Agricultural Sciences, University of Naples Federico II, Naples, Italy; ^2^Department of Vegetable Crops, Agricultural Research Institute, Nicosia, Cyprus

**Keywords:** anti-nutrients, chemical eustressor, functional quality, floating system, micronutrients, mild salt stress, nutrient solution management, stress response

## Abstract

Closed soilless cultivation systems (SCS) support high productivity and optimized year-round production of standardized quality. Efficiency and precision in modulating nutrient solution composition, in addition to controlling temperature, light, and atmospheric composition, renders protected SCS instrumental for augmenting organoleptic and bioactive components of quality. Effective application of eustress (positive stress), such as moderate salinity or nutritional stress, can elicit tailored plant responses involving the activation of physiological and molecular mechanisms and the strategic accumulation of bioactive compounds necessary for adaptation to suboptimal environments. For instance, it has been demonstrated that the application of salinity eustress increases non-structural carbohydrates and health-promoting phytochemicals such as lycopene, β-carotene, vitamin C, and the overall phenolic content of tomato fruits. Salinity eustress can also reduce the concentration of anti-nutrient compounds such as nitrate due to antagonism between nitrate and chloride for the same anion channel. Furthermore, SCS can be instrumental for the biofortification of vegetables with micronutrients essential or beneficial to human health, such as iodine, iron, selenium, silicon, and zinc. Accurate control of microelement concentrations and constant exposure of roots to the fortified nutrient solution without soil interaction can maximize their uptake, translocation, and accumulation in the edible plant parts; however, biofortification remains highly dependent on microelement forms and concentrations present in the nutrient solution, the time of application and the accumulation capacity of the selected species. The present article provides an updated overview and future perspective on scientific advances in SCS aimed at enhancing the sensory and bioactive value of vegetables.

## Soilless Means for Improving Sensory and Functional Quality of Vegetables

The productivity of agricultural production systems is unprecedentedly challenged by projections for global population increase, by climate change and by shortage of the fundamental natural resources of water and arable land. In the case of vegetable crops, which contribute significant nutritive and bioactive value to the human diet, maximal productivity is attained under controlled environments where production may expand vertically and temperature, light, nutrient supply, and atmospheric composition are controlled (Gruda, 2005, 2009). In particular, the technological advancement of closed soilless (hydroponic) cultivation systems (SCS; e.g., nutrient film, floating, and pot- and sacs-systems) based on recirculating nutrient solutions has maximized productivity per unit area and notably in terms of water use efficiency, by maximizing root contact with the nutrient source while minimizing evaporation and nutrient runoff (Treftz and Omaye, 2016). Despite the high capital investment and technological proficiency required for managing soilless systems, their expansion is propelled by the efficacy of optimized year-round production and standardized product quality irrespective of locality.

Besides the pressing issue of global food security, demand for high quality horticultural products is also on the rise, driven by the growing interest of society in fresh products of high organoleptic, nutritional, and functional quality. The quality of fresh horticultural commodities has been recently defined as “*a dynamic composite of their physicochemical properties and evolving consumer perception, which embraces organoleptic, nutritional and bioactive components*” (Kyriacou and Rouphael, 2018). Extrinsic characteristics of product quality are strongly influenced by socioeconomic and marketing factors which formulate consumer perception and generate quality prototypes. Despite the continued growth of the hydroponic industry consumers at large hold a negative bias toward SCS products which they consider artificial, less tasty, and of lower nutritional quality (Schnitzler and Gruda, 2002), just as organically grown fruits and vegetables are generally hailed as healthier and safer (Orsini et al., 2016). Nevertheless, it is apparent that key secondary metabolites which form the basis of functional quality in horticultural products can be modulated by appropriate management of SCS components. Exposure to biotic and abiotic stress underlies the superior nutritional quality often observed in organically grown products, since stress response entails the activation of physiological and molecular mechanisms necessary for adaptation to suboptimal environments, such as the biosynthesis of secondary metabolites (e.g., ascorbate, tocopherols, carotenoids, and glucosinolates; Orsini et al., 2016). Soilless systems can facilitate the precise application of an eustress (positive stress), such as moderate salinity or nutritional stress, through precise management of the concentration and composition (cationic and anionic proportions or single ions) of the nutrient solution, and thus may constitute a practical and effective means for improving the nutritional value of vegetables and for reducing the accumulation of anti-nutrient compounds, such as nitrates (Colla et al., 2018; Rouphael and Kyriacou, 2018; Rouphael et al., 2018a,b). Soilless culture can also be instrumental in the biofortification of edible plant portions with essential and/or beneficial micronutrients to human health. Biofortification with essential or beneficial micronutrients may constitute an effective means for supplying the human diet with iodine (I), selenium (Se), zinc (Zn), and silicon (Si) (White and Broadley, 2005). The present article provides an updated overview and future perspective on scientific advances in soilless cultivation aimed at enhancing the sensory and bioactive quality of vegetables through nutrient solution management and applications aimed at biofortification.

## Salinity Eustress and Macronutrient Management for Enhancing Nutritional Quality of Hydroponically Grown Vegetables

Excessive concentration of sodium chloride (NaCl) in irrigation water and agricultural soils disturbs physiological processes in vegetable crops, leading to stunted growth and yield decline (Rouphael et al., 2017, 2018b). However, recent scientific reviews have indicated that vegetable crops may exhibit tailored responses to the application of eustress, such as mild to moderate salinity, as a result of stress-induced reshuffling of plant metabolism and strategic accumulation of bioactive compounds against suboptimal conditions (Kyriacou and Rouphael, 2018). Vegetable crops can synthesize a broad range of secondary metabolites to counteract oxidative damage and to scavenge reactive oxygen species (ROS) elicited by environmental stressors (Orsini et al., 2016). These health-promoting phytochemicals, abundant in stressed plants, can enrich the functional quality of fresh vegetables to the benefit of human diet (Khanam et al., 2012; Kyriacou and Rouphael, 2018).

Multiple studies have reported increase in the bioactive content of vegetables triggered by mild to moderate NaCl concentrations in the nutrient solution (Tzortzakis, 2009, 2010; Rouphael et al., 2018a,b). Although under soil conditions this technique for improving product quality poses a high risk of plant overstress (Hidaka et al., 2008), soilless culture may be an effective tool for modulating secondary metabolites without curbing growth and yield, through proper management of the nutrient solution’s composition (Schwarz et al., 2009; Tomasi et al., 2015). Several studies have demonstrated that NaCl in the nutrient solution raises the levels of sugars, organic acids, and amino acids in several vegetable crops, like tomato (Zushi and Matsuzoe, 2015; Moya et al., 2017), pepper (Marin et al., 2009), melon (Rouphael et al., 2012b), watermelon (Colla et al., 2006), eggplant (Savvas and Lenz, 1996), lettuce (Sakamoto et al., 2014), and cauliflower (Giuffrida et al., 2017) thereby improving their organoleptic quality. The salt-induced osmoregulatory mechanism in hydroponically grown vegetables involves the biosynthesis of specific osmolytes (sugars, minerals, and amino acids such as proline and GABA) believed to function as osmoprotectants by counterbalancing the increase in vacuolar osmotic potential caused by the toxic accumulation of sodium and chloride ions (Hasegawa et al., 2000).

The application of salinity eustress may also affect health-promoting phytochemicals. For instance, increasing soilless nutrient solution electrical conductivity (EC) from 3 to 6.5 dS m^-1^ (Krauss et al., 2006), from 2.4 to 4.5 dS m^-1^ (Wu et al., 2004), and from 2.2 to 4.5 dS m^-1^(Moya et al., 2017) increased the lycopene, β-carotene, vitamin C, and total phenolic content of tomato. Giuffrida et al. (2017), showed that the functional quality of hydroponically grown cauliflower in response to moderate salt stress may depend on interactive variables, such as duration of exposure and plant phenological stage at the time of exposure (e.g., salinity stress applied constantly throughout the cultivation cycle or at the onset of flowering), with neoglucobrassicin concentration found two-fold higher in cauliflower heads supplied with saline nutrient solution (4 dS m^-1^) compared to the non-saline (EC 2 dS m^-1^) control treatment. Beneficial effects of mild to moderate salinity on nutritional and bioactive value was also reported for hydroponically grown leafy greens (Kim et al., 2008; Colla et al., 2013; Klados and Tzortzakis, 2014; Bonasia et al., 2017; Ntatsi et al., 2017; Petropoulos et al., 2017). For instance, Petropoulos and co-workers reported that increasing the EC from 1.8 to 6.0 dS m^-1^ increased ascorbic acid as well as α-tocopherol levels in spiny chicory. Similarly, Colla et al. (2013) and Bonasia et al. (2017) showed that raising the EC from 2.5 to 3.5 dS m^-1^ increased antioxidant compounds in wild rocket, and from 2.0 to 5.8 dS m^-1^ it improved the antioxidant activity, chlorogenic acid, cynarin, and luteolin levels in leaves of artichoke and cardoon grown in a floating system. However, response to NaCl is cultivar-dependent and the choice of the cultivar is critical for achieving the desired effects (Borghesi et al., 2011; Dominguez-Perles et al., 2011; Colla et al., 2013). Several workers have reported negative effects or no significant effects in response to NaCl application. Increasing the nutrient solution EC above 4.4 dS m^-1^ decreased lycopene and β-carotene content in tomato (De Pascale et al., 2001). Similarly, Petersen et al. (1998) and Bonasia et al. (2017) observed a decrease of ascorbic acid in tomato and wild rocket at EC 9.0 and 4.5 dS m^-1^, respectively. Presumably the antioxidant system of salt-stressed plants does not effectively support ROS scavenging after the stress threshold for maintaining growth is exceeded (Rouphael et al., 2018a,b), whereas leaf area reduction in salt-sensitive cultivars can also modify fruit temperature and halt the synthesis of bioactive compounds (Dorais et al., 2008). Finally, salinity eustress can reduce nitrate accumulation in SCS leafy vegetables due to antagonism between nitrate and chloride for the same anion channel (Rubinigg et al., 2003). Vegetable nitrate remains of high interest to regulators due to possible effects on human health, while it also imparts vegetables a bitter taste (Colla et al., 2018). Borgognone et al. (2016) reported that minimizing nitrate supply in floating raft culture by partial substitution of calcium nitrate with calcium chloride increased phenols and flavonoids and lowered nitrates in cardoon leaves without affecting yield.

Although most published articles concerning the positive effects of nutrient solution EC on nutritional, organoleptic, and functional quality of soilless vegetables were based on greenhouse experiments in which NaCl was the predominant salt, several studies have shed light on the effects of salinity induced by macronutrients. For instance, Fallovo et al. (2009) determined the effects of macronutrient solution concentration (2, 18, 34, 50, or 66 mequiv L^-1^, corresponding to an EC of 0.3, 1.2, 2.0, 2.8, and 3.6 dS m^-1^, respectively) during the spring and summer seasons on leafy lettuce (*Lactuca sativa* L. var. *acephala*) grown in a floating system. The authors reported a linear decrease in qualitative characteristics (glucose, fructose, proteins, total carbohydrates, and starch contents) in response to an increase in the nutrient solution concentration from 2 to 66 mequiv L^-1^. Similarly, Rouphael et al. (2012a) showed that raising the macronutrient solution concentration from 4 to 68 mequiv L^-1^ in floating raft culture increased biomass production but deteriorated leaf functional quality in both cardoon and artichoke by decreasing key polyphenols such as caffeic acid, chlorogenic acid, cynarin, and luteolin. Moreover, the management of the cationic proportions (K/Ca/Mg) in the nutrient solution facilitated by soilless culture has been also demonstrated as an effective tool for enhancing nutritional quality of fruit vegetables (Fanasca et al., 2006a). A high proportion of K in the nutrient solution caused a significant increase in tomato soluble solids and lycopene contents irrespective of cultivar (‘Corfu’ or ‘Lunarossa’ – standard or high-pigment cultivar), whereas high concentration of Mg improved the hydrophilic antioxidants (caffeic acid) and the total antioxidant capacity of the high-pigment ‘Lunarossa’ hybrid (Fanasca et al., 2006b). Synthesis and accumulation of the abovementioned antioxidant compounds in response to high Mg supply might relate to the increased activity of key enzymes such as glutamine synthetase that regulate ammonia assimilation and detoxification of plant tissues (Marschner, 2012).

## Soilless Biofortification of Vegetables with Essential and Beneficial Micronutrients

Biofortification of vegetables with essential and non-essential beneficial micronutrients caters to the demand for healthier diet and the need to address human micronutrient deficiency, known as “*hidden hunger*” (White and Broadley, 2005; Carvalho and Vasconcelos, 2013). However, the window between biofortification and toxicity effect is often quite narrow. Applications aiming at the accumulation of health-supporting micronutrients must be adjusted to avoid detrimental effects on plant growth (Rouphael et al., 2018a). Moreover, biofortification may depend upon several interacting factors, such as genotype, chemical form, application rate, and environmental and growing conditions (Tomasi et al., 2015).

Selenium and iodine have been particularly investigated since they are beneficial though non-essential microelements for human health. Uptake is higher when supplied in SCS nutrient solution where Se and I concentrations can be accurately controlled, as opposed to side-dressing of soil crops or foliar applications (Wiesner-Reinhold et al., 2017). Constant exposure of the root system to fortified nutrient solution and absence of micronutrient–soil interaction make SCS more efficient, thus maximizing uptake, translocation, and accumulation of these elements in the edible parts (Wiesner-Reinhold et al., 2017). However, micronutrient accumulation is highly dependent on the elemental concentration in the soilless solution, the time of application and the accumulation capacity of the selected species. For instance, Signore et al. (2018) reported that iodine biofortification of carrots at 50 mg L^-1^ through the hydroponic solution reached cumulative levels toxic on the plants compared to foliar applications both under open-field and greenhouse conditions at the same rate. On the other hand, low rates (0.5–1.5 mg L^-1^) of selenium in the nutrient solution increased Se concentration in several horticultural commodities such as spinach, lettuce, and basil without inducing toxic effects (Zhu et al., 2004; Malorgio et al., 2009; Ramos et al., 2011; Ferrarese et al., 2012; Puccinelli et al., 2017). Zhu et al. (2003), reported that biofortification with I in solution culture is easily feasible both with iodide (I^-^) and iodate (${\text{IO}}_{3}^{-}$), as the application of I at rates from 0.13 to 12.7 mg L^-1^ effectively fortified spinach with iodine. Similarly, Blasco et al. (2008) showed that the most appropriate rate of I^-^ in lettuce soilless culture is 5.1 mg L^-1^ or lower, whereas ${\text{IO}}_{3}^{-}$ concentration of 1.3 to 30.5 mg L^-1^ achieved foliar accumulation of I without detriment to yield. Kiferle et al. (2013); Li et al. (2017), and Smoleń et al. (2018) indicated that also fruit vegetables and tuber crop such as tomato, pepper, and potato grown in soilless culture can be targeted for I and/or Se biofortification. Another important factor influencing Se and I accumulation in vegetables is their chemical forms. Vegetable species exposed to selenate (O_4_Se^-2^) rather than selenite (O_3_Se^-2^) and to I^-^ rather to ${\text{IO}}_{3}^{-}$ can accumulate more Se and I in leaf or fruit tissues. This is because selenate is taken up actively by the more efficient sulfate transporter compared to the passive phosphate transporter used for taking up selenite (Wiesner-Reinhold et al., 2017). Uptake of I^-^ was much higher than ${\text{IO}}_{3}^{-}$ since the latter form should be reduced to I^-^ before uptake, thus reducing bioavailability to vegetable crops (Blasco et al., 2008).

According to White and Broadley (2005), concentrations of 0.1–0.7 mg Zn g^-1^ dry weight can be achieved in leafy vegetables with no detriment to yield, making Zn biofortification of leafy greens a potential tool for enhancing dietary Zn intake (White et al., 2018). Adequate Zn addition in the nutrient solution (5.2–6.5 mg L^-1^) allowed biofortification of *Brassica oleracea* coupled with significant synthesis and accumulation of amino acids (Arg, Asp, Glu, Gln, Hys, Lys, Phe, and Trp) while maintaining optimal growth (Barrameda-Medina et al., 2017). In addition, the production of Fe-enriched leafy vegetables such as lettuce using hydroponics is feasible, since the increase of Fe concentration in the nutrient solution 6 h before harvest resulted in significant increase of foliar Fe content without affecting yield (Inoue et al., 2000). Moreover, effective Si biofortification of soilless crops of basil, chicory, mizuna, purslane, Swiss chard, and tatsoi was demonstrated with SiO_2_ supplementation of the nutrient solution at 50–100 mg L^-1^ with no detrimental effect on crop productivity (D’Imperio et al., 2016).

## Conclusion and the Challenges Ahead

The demand for global food security under increasing biotic and abiotic pressures exacerbated by climate change makes protected cultivation of vegetable crops an inevitable necessity. Technological progress in the management of SCS drives productivity and mitigates high initial infrastructural costs. Moreover, flexibility and precision in modulating nutrient solution composition, in addition to controlling temperature, light, and atmospheric composition, renders SCS systems instrumental in targeting organoleptic and bioactive components of quality thus addressing demand for improved vegetable quality. The effective application of eustress, such as mild to moderate salinity or nutritional stress, can elicit targeted plant responses through the activation of physiological and molecular mechanisms and the strategic accumulation of bioactive compounds necessary for adaptation to suboptimal environments. Salinity eustress has been demonstrated to augment organoleptic components of quality such as soluble carbohydrates, and health-promoting phytochemicals such as lycopene, β-carotene, vitamin C, and polyphenols in vegetables; moreover, it may curb anti-nutrients such as nitrate owing to nitrate-chloride antagonism for uptake. Understanding the molecular and physiological mechanisms elicited by controlled plant eustress and those facilitating micronutrient uptake in interaction with genotype and environmental conditions will usher horticultural science into the era of tailoring superior sensory and functional quality vegetables.

Furthermore, SCS can facilitate the effective biofortification of vegetables with micronutrients essential or beneficial to human health, such as iodine, iron, selenium, silicon, and zinc. Accurate control of microelement concentrations and constant exposure of roots to the fortified nutrient solution without soil interaction can maximize their uptake, translocation, and accumulation in the edible plant parts. Biofortification remains, however, highly dependent on microelement forms and concentrations present in the nutrient solution, the duration of targeted applications, the developmental stage of plants, and the accumulation capacity of the selected species. These potentially interacting factors pose future challenges for research before SCS biofortification applications become effective tools for addressing nutrient deficiencies in human diet.

## Author Contributions

YR and MK had the original idea of eustress and biofortification of soilless vegetables and were both involved in writing the article.

## Conflict of Interest Statement

The authors declare that the research was conducted in the absence of any commercial or financial relationships that could be construed as a potential conflict of interest.
